# Distribution, Metabolism, and Recovery of Resin Acids in the Intestine and Tissues of Broiler Chickens in a Feeding Trial With Tall Oil Fatty Acid-Supplemented Diets

**DOI:** 10.3389/fvets.2020.00437

**Published:** 2020-07-28

**Authors:** Juha Apajalahti, Kirsi Vienola, Kari Raatikainen, Hannele Kettunen, Juhani Vuorenmaa

**Affiliations:** ^1^Alimetrics Research Ltd, Espoo, Finland; ^2^Hankkija Ltd, Hyvinkää, Finland

**Keywords:** tall oil fatty acids, resin acids, resin acid conjugates, bacterial deconjugation, broiler chickens

## Abstract

Tall oil fatty acids (TOFA) are novel, health-improving feed ingredients which have been shown to improve the performance of broiler chickens. TOFA contains resin acids, the suggested key components for its beneficial effects. For product safety, possible accumulation of TOFA components in tissues consumed by end-users is an issue of major importance. Wheat-soy-based diets with an indigestible marker and TOFA at 0, 750 and 3,000 g/t were fed to broiler chickens for 5 weeks; 11 replicate pens/treatment. Deposition of resin acids was assessed by analyzing jejunal tissue, breast muscle, abdominal fat, blood, liver, bile, and digesta along the intestinal tract at the end of the 35-day trial. Both free and conjugated resin acids were quantified. With TOFA 3,000 g/t diet, 30% of ingested resin acids could not be recovered from jejunal digesta. Also, a proportion representing 45% of resin acids fed were in conjugated form and thus had already re-entered the intestine from the bile duct. This means that at least 75% of resin acids ingested had become absorbed in, or proximal to jejunum. Recovery of resin acids in excreta was 45 and 70% when TOFA was fed at 750 and 3,000 g/t, respectively. Based on recovery data, of the estimated 1,087 mg of resin acids ingested by birds on the high TOFA dose during their lifetime, about 330 mg was unaccounted for. In analysis of jejunal tissue, blood, liver, bile, breast muscle, and abdominal tissue, <1 mg of resin acids was found after the 35-day trial when TOFA at the 4-fold the recommended dose was fed. It is likely that the host or microbiota mineralized or converted one-third of resin acids to a form that escaped analysis. TOFA at 3,000 g/t dose caused no detectable adverse effects in broiler chickens. Based on analysis of breast meat and liver, the common edible tissues, a human consumer would ingest <100 μg of resin acids in a single meal. That is one-thousandth of the dose shown to be harmless in rodents. Thus, unintentional exposure of human consumers to resin acids is marginal, and posed no safety concerns.

## Introduction

The ban on antibiotic growth promoters in an increasing number of countries worldwide has given rise to an intensive search for new products to improve animal performance and health. The most common product categories are organic acids, live-fed microbes (probiotics), oligosaccharides (prebiotics), and many plant-derived products. One new additive used to improve performance and health in production animals is tall oil fatty acids (TOFA), a high-volume side-product from the wood processing industry. It is derived from coniferous trees in the Kraft process, where acylglycerols are hydrolyzed under alkaline conditions into free fatty acids and glycerol, then acidified and eventually distilled to produce the final TOFA product (1).

The raw material for the TOFA used in the present study originated from northern coniferous trees, mainly Scots pine (*Pinus sylvestris*) and Norway spruce (*Picea abies*) grown in Finland. Besides long-chain saturated and unsaturated fatty acids (~90%), the product also contained free resin acids (8.6%). Resin acids are tricyclic diterpenoids, the most abundant of which in the TOFA product used here were abietic acid, dehydroabietic acid, and palustric acid. Together, these three specific acids accounted for 53.5% of total resin acids in the product.

The interest in using TOFA and resin acids in animal diets increased substantially when TOFA products were shown to improve body weight gain and feed conversion efficiency in broiler chickens (2–4). The mechanism explaining the positive effects is not known in detail. However, TOFA has been shown to inhibit *in vitro* growth of some bacteria, especially *Clostridium perfringens* (4). It is also reported to modulate the immune system of broiler chickens, by suppressing both duodenal inflammatory T cell infiltration and expression of collagen-degrading matrix metalloproteinases (5, 6). During the past year, the role of different TOFA compounds has been intensively studied. The results suggest that resin acids may be the most important group of compounds mediating positive effects of TOFA on animal performance (7).

Whatever the underlying mechanism for the beneficial growth effects in broiler chickens, it is important for product safety to know whether any components of TOFA products accumulate in tissues consumed by end-users. While the fate and metabolism of long-chain fatty acids in animals have been extensively studied (8, 9), the fate of ingested resin acids is poorly understood. There are no prior reports showing the detailed effects of resin acids in monogastric production animals in tissue level. However, their metabolism in fish has been studied in research investigating how the discharge of resin acid-containing wastewaters from wood processing affects fish living in recipient waterways (10, 11). In those studies, resin acids have been in water-soluble form and the entry to fish is assumed to be primarily through gills. With rodents, only toxicity studies with resin acids have been reported. Although rats are different from poultry and pigs, at least the exposure to resin acids comes through the same oral route. These studies suggest that rodents can tolerate relatively high doses of resin acids (12–14).

In the present study, the main objective was to investigate the distribution of resin acids in the digestive tract and tissues of broiler chickens when a commercial TOFA product was added in the diet at the recommended and 4-fold the recommended dose. Using an indigestible marker compound in the diet, the proportion of in-feed resin acids excreted with feces was also assessed. With the experimental arrangement used it was also possible to analyze the extent to which accumulation of resin acids in tissues took place during the 5-week trial.

## Materials and Methods

### Animals and Housing

A 35-day feeding trial with broiler chickens, in accordance with EU Directive 2010/63/EU, was conducted at the research facility of Alimetrics Ltd in Southern Finland. The trial was started in January 2018. Newly hatched chicks were obtained from a commercial hatchery (DanHatch, Mynämäki, Finland) and were not given vaccinations or commercial inoculants. In the trial, 15 male broilers of breed Ross 308 (Aviagen) were allocated in each floor pen. There were 3 dietary treatments each fed to 11 replicate pens; in total 33 pens and 495 birds.

The temperature of the house was raised to 32°C 2 days before the chicks arrived. One-day-old chicks were randomly allocated to 33 open floor pens of 1.125 m^2^, bedded with wood shavings litter. Luminosity was adjusted to 20 lux and air humidity to 60%. Brooder lamps were adjusted to provide extra heating during the first week, and the temperature was gradually decreased to 22°C during the first 2 weeks of the trial. Temperature, ventilation, and humidity were monitored continuously throughout the trial. From day 1, the hours of darkness were increased daily by 1 h from 24 h light until the light-dark cycle was 18 h light and 6 h darkness daily. Feed and water were freely available at all times. The weight of birds and feed consumption per pen were measured on days 0, 14, 21, and 35. Dead birds and birds euthanized because of health problems were weighed and daily mortality was recorded.

### Dietary Treatments

The basal starter and grower diets were wheat-soy based pelleted feeds, the composition of which are shown in Table 1. Two-phase feeding was used: the starter diet was fed on days 0–14 and the grower diet on days 14–35. The starter diets were prepared as 2 mm crushed pellets and the grower diets as 4 mm pellets. The dietary treatments employed in each trial and the results of diet analysis are shown in Table 2. In addition to the control, treatments with TOFA at two doses were used. The TOFA was mixed with sunflower oil before inclusion in the treatments and it replaced a corresponding amount of sunflower oil in the diet. The test TOFA product used was a commercial product (Progres^TM^) obtained from Hankkija Ltd (Hyvinkää, Finland) and produced at the tall oil refinery of Forchem Ltd (Rauma, Finland). The test TOFA product contained about 90% free fatty acids (of which 50–55% various linoleic acids, 25–30% oleic acid, and 6–8% pinolenic acid) and 8.6% resin acids. Titanium dioxide (TiO_2_) was added to all diets at 3 kg/t as a digestibility marker. All feeds were manufactured by Alimetrics Ltd, Finland.

**Table 1:** Components of basic diets used in the trial.

**Added ingredients (g/kg)**	**Starter diet**	**Grower diet**
Wheat	571.5	675.1
Soybean meal	340.0	253.0
Sunflower oil	41.0	3.2
Monocalcium phosphate	16.5	15.2
Limestone	16.7	12.5
Sodium chloride	4.1	3.9
Mineral premix^a^	2.0	2.0
Vitamin premix^b^	2.0	2.0
DL-methionine	2.7	2.2
L-lysine	2.5	2.1
Threonine	1.0	0.0
**Calculated nutrient concentrations**	**Starter diet**	**Grower diet**
Metabolizable energy (MJ/kg)	12.30	12.51
Crude protein	231.29	201.81
Lysine	13.38	10.98
Methionine	5.97	5.11
Threonine	9.37	7.13
Methionine + cysteine	9.54	8.42
Calcium	10.35	8.32
Non-phytate phosphorus	4.27	3.94

a *composition: calcium 296.9 g/kg, zinc 32.5 g/kg, manganese 25.0 g/kg, iron 12.5 g/kg, copper 4.0 g/kg, iodine 225 mg/kg, selenium 100 mg/kg*.

b *composition: calcium 331.3 g/kg, all-rac-α-tocopheryl acetate 30.0 g/kg, niacin 20.1 g/kg, panthotenic acid 7.51 g/kg, riboflavin 3.0 g/kg, pyridoxine 2.01 g/kg, retinol 1.8 g/kg, menadione 1,505 mg/kg, thiamine 1,257 mg/kg, folic acid 504 mg/kg, biotin 75.0 mg/kg, cholecalciferol 56.3 mg/kg, cobalamin 12.5 mg/kg*.

**Table 2:** Analyzed composition of the starter and grower diets fed to broiler chickens.

	**Starter diets**			**Grower diets**			
**Concentration/feed DM**	**Control**	**TOFA 750 g/t**	**TOFA 3,000 g/t**	**Control**	**TOFA 750 g/t**	**TOFA 3,000 g/t**	**SE**
DM, %	90.6	91.1	91.0	89.6	89.9	89.8	0.10
TiO_2_, kg/t	2.82	2.90	2.83	3.23	3.08	3.18	0.11
AA, g/t	0.0	31.6	101	0.0	32.2	100	0.79
DHA, g/t	0.0	14.1	47.6	0.0	15.0	47.4	0.68
PA, g/t	0.0	6.3	19.4	0.0	6.2	18.5	0.23
AA + DHA + PA, g/t	0.0	52.0	168	0.0	53.4	166	1.61
Calc. total RA, g/t^a^	0.0	97.2	314	0.0	99.8	310	3.01
Crude protein, kg/t^b^	249	251	258	219	202	211	–
Crude fat, kg/t^b^	65	69	69	61	60	59	–
Crude fiber, kg/t^b^	20	19	18	20	21	19	–
Ash, kg/t^b^	76	79	79	71	70	70	–
N-free extractives, kg/t^b^	589	582	576	629	646	641	–

*TOFA, tall oil fatty acids; AA, abietic acid; DHA, dehydroabietic acid; PA, palustric acid; RA, resin acids; SE, standard error*.

a *calculation based on the percentage of AA + DHA + PA in total resin acids in TOFA (53.5%), as reported by the manufacturer*.

b *proximate analysis outsourced to Eurofins*.

The feeds were subjected to proximate analysis, and analysis of TiO_2_ and major resin acids. The results indicated that nutritional values were close to the target and nearly identical in different starter and grower diets (Table 2). The target TiO_2_ dose was 3 kg/t. The analyzed values in starter diets ranged between 2.82 and 2.90 kg/t and those in grower diets between 3.08 and 3.23 kg/t. Resin acid analysis concentrated on three major individual acids (abietic, dehydroabietic, and palustric). The analysis showed that the combined concentration of the three acids were 52.0 and 53.4 g/t, respectively, for the starter and grower diet supplemented with 750 g/t TOFA, and 168 and 166 g/t, respectively, for the starter and grower diet supplemented with 3,000 g/t TOFA. According to the TOFA manufacturer's specification, the three acids analyzed represented 53.5% of total resin acids in the product. Based on this information, the total resin acid content of the diets was calculated to be ~98 and 312 g/t for the diets supplemented with 750 and 3,000 g/t TOFA, respectively (Table 2). The figures obtained from such arithmetic procedure in this paper are referred to as “total calculated resin acids” and it is based on the assumption that all resin acids in TOFA act in live animal similar to the dominating ones abietic and dehydroabietic acid.

### Sampling

On day 34 of the trial, a piece of plastic film was spread out in each pen on the litter material for excreta collection. After 3 h, the plastic films were removed and excreta from each pen were carefully transferred to a glass jar and frozen until analysis. On day 35, two birds from each pen were euthanized by cervical dislocation. The abdominal cavity was opened, the small intestine removed, and digesta from jejunum and ileum snap-frozen in dry ice and stored frozen until analysis of resin acids and TiO_2_. In addition, samples of jejunal tissue, breast muscle tissue, abdominal fat, blood, liver, and bile were taken from the birds on the control diet and birds on the diet with the higher dose of TOFA, and analyzed for resin acids. Blood samples were taken with cardiac puncture immediately after euthanasia, mixed with heparin, and centrifuged at 1,500 × g for 10 min for plasma preparation. The rationale for selecting specific sample types for analysis was to get information firstly on resin acid conjugation and passage through enterohepatic circulation and secondly on resin acid concentration in some common edible tissues. Fecal sampling took place 1 day before the animals were sacrificed since it was not possible to collect feces and sacrifice animals for tissue sampling at the same time without compromising both sampling procedures. All tissue samples were frozen immediately and stored at −20°C until analysis.

### Analysis Methods

#### Resin Acids

##### General procedure for analysis of all sample matrices

Samples were thawed and thoroughly homogenized. For analysis of total (free + conjugated) resin acid content, representative samples were weighed and mixed with 1.5–6.0M KOH (depending on the tissue type; see details below), followed by incubation in a water bath at 75°C for 2 h. A previously published alkaline hydrolysis procedure was used to liberate resin acids from glucuronide and sulfate conjugates (15, 16). After hydrolysis, the samples were cooled in ice-water and neutralized to pH 7–8 with 3M H_3_PO_4_. In the analysis of free resin acids, the samples were not hydrolyzed but mixed with a pH-neutral mixture of KOH and H_3_PO_4_, resulting in a mixture with equal ion strength as the samples analyzed for total resin acids.

Resin acids were then extracted for 30 min with a 1:4 mixture of methanol and ethyl acetate containing nonadecanoic acid as an internal standard. After centrifugation, the organic phase was collected and a defined subsample of that evaporated to dryness (see exact volumes for each sample type below). The residue was then dissolved in a solution with 1 volume of N,O-bis-trimethylsilyl-trifluoroacetamide with 1% trimethylchlorosilane and 1 volume of pyridine. The analyte was derivatized to the respective trimethylsilyl ester by heating the sample at 60°C for 30 min. Matrix-matched standards were used in calibration. An Agilent 7890A gas chromatograph equipped with 5975C mass detector was used in the analyses. The column was HP-5MS (60 m × 250 μm × 0.25 μm) and data were collected with single ion mode. Feed samples were analyzed and quantified for abietic, dehydroabietic, and palustric acid. It turned out to be difficult to quantify palustric acid in tissues since its proportion of total resin acids was low as compared to abietic and dehydroabietic acid, and, in the difficult sample matrices no baseline separation was achieved. Therefore, we report only abietic and dehydroabietic acid concentrations in samples of animal origin.

##### Analysis of feed, digesta, and liver samples

A representative 1.5 g sample was hydrolyzed with 2 mL of 1.5M KOH and neutralized with 0.5 mL of 3M H_3_PO_4_. Ten milliliter of organic extraction solution was used, of which 0.5 mL was collected, evaporated to dryness and derivatized with 150 μL of the silylation reagent.

##### Analysis of meat samples

A representative 5.0 g sample was hydrolyzed with 3 mL of 3M KOH and neutralized with 1.5 mL of 3M H_3_PO_4_. In this case, 25 mL of organic extraction solution was used, of which 1.0 mL was collected, evaporated to dryness and derivatized with 200 μL of the silylation reagent.

##### Analysis of adipose tissue samples

A representative 0.5 g sample was hydrolyzed with 1 mL of 3M KOH and neutralized with 0.5 mL of 3M H_3_PO_4_. Ten milliliter of organic extraction solution was used, of which 0.5 mL was collected, evaporated to dryness and derivatized with 150 μL of the silylation reagent.

##### Analysis of jejunal tissue samples

A representative 0.5 g sample was hydrolyzed with 0.4 mL of 6M KOH and neutralized with 0.4 mL of 3M H_3_PO_4_. In this case, 4.4 mL of organic extraction solution was used, of which 2.5 mL was collected, evaporated to dryness and derivatized with 200 μL of the silylation reagent.

##### Analysis of blood plasma samples

A 0.4 mL sample was hydrolyzed with 0.1 mL of 6M KOH and neutralized with 0.1 mL of 3M H_3_PO_4_. One milliliter of organic extraction solution was used, of which 0.5 mL was collected, evaporated to dryness and derivatized with 150 μL of the silylation reagent.

##### Analysis of bile samples

A 0.2 mL sample was hydrolyzed with 0.1 mL of 6M KOH and neutralized with 0.1 mL of 3M H_3_PO_4_. One milliliter of organic extraction solution was used, of which 0.5 mL was collected, evaporated to dryness and derivatized with 150 μL of the silylation reagent.

#### TiO_2_

The method used for the analysis of TiO_2_ in feed, digesta, and excreta followed a previously published protocol (17). The method involves sample combustion in a furnace, titanium (Ti) dissolution in hot sulfuric acid (H_2_SO_4_), and finally reaction with hydrogen peroxide (H_2_O_2_). The intensely colored Ti-H_2_O_2_ complex was measured quantitatively with UV/VIS spectroscopy.

#### Dry Matter Content

For dry matter determination, samples were weighed, dried at 105°C for 12 h, and weighed again.

#### Statistical Analysis

The data were first subjected to one-way ANOVA using the IBM SPSS Statistics Subscription (Build 1.0.0.1327). ANOVA *P*-values lower than 0.05 were considered significant. The data were then subjected to either Tukey's test or Students' *t*-test, as specified in the legend of each table and figure.

## Results

### Effect of Tall Oil Fatty Acids on Performance of Broiler Chickens

The broiler chicken groups were fed the control diet or a diet supplemented with TOFA at 750 or 3,000 g/t for 5 weeks. In this trial, none of the treatments had any effect on performance parameters (Table 3).

**Table 3:** Effect of diets containing tall oil fatty acids on performance of broiler chickens.

**Parameter**	**Control**	**TOFA 750 g/t**	**TOFA 3,000 g/t**	**SE^a^**	***P*-value^b^**
Day 0–14					
Initial BW (g)	38.9	38.6	38.8	0.19	0.450
BWG (g)	507	495	506	7.12	0.424
FC (kg/pen)	8.7	8.5	8.7	0.11	0.525
FCR	1.16	1.16	1.18	0.01	0.331
Mortality (%)	1.8	1.2	3.0	0.93	0.390
Mortality-corrected FCR	1.15	1.15	1.16	0.00	0.334
Day 0–21					
BWG (g)	1,023	1,003	1,019	14.15	0.587
FC (kg/pen)	20.4	20.0	20.3	0.29	0.598
FCR	1.36	1.37	1.38	0.01	0.429
Mortality (%)	1.8	2.7	3.0	1.20	0.526
Mortality-corrected FCR	1.35	1.36	1.37	0.01	0.358
Day 0–35					
BWG (g)	2,694	2,637	2,666	33.74	0.504
FC (kg/pen)	59.9	59.1	59.8	1.15	0.882
FCR	1.60	1.55	1.58	0.02	0.349
Mortality (%)	6.1	3.0	4.2	1.67	0.468
Mortality-corrected FCR	1.54	1.54	1.55	0.01	0.270

*TOFA, tall oil fatty acids; BW, body weight; BWG, body weight gain; FC, feed consumption; FCR, feed conversion ratio*.

a *pooled standard error*.

b *P-value from ANOVA*.

### Passage of Resin Acids Through the Intestinal Tract

**Figure 1:**
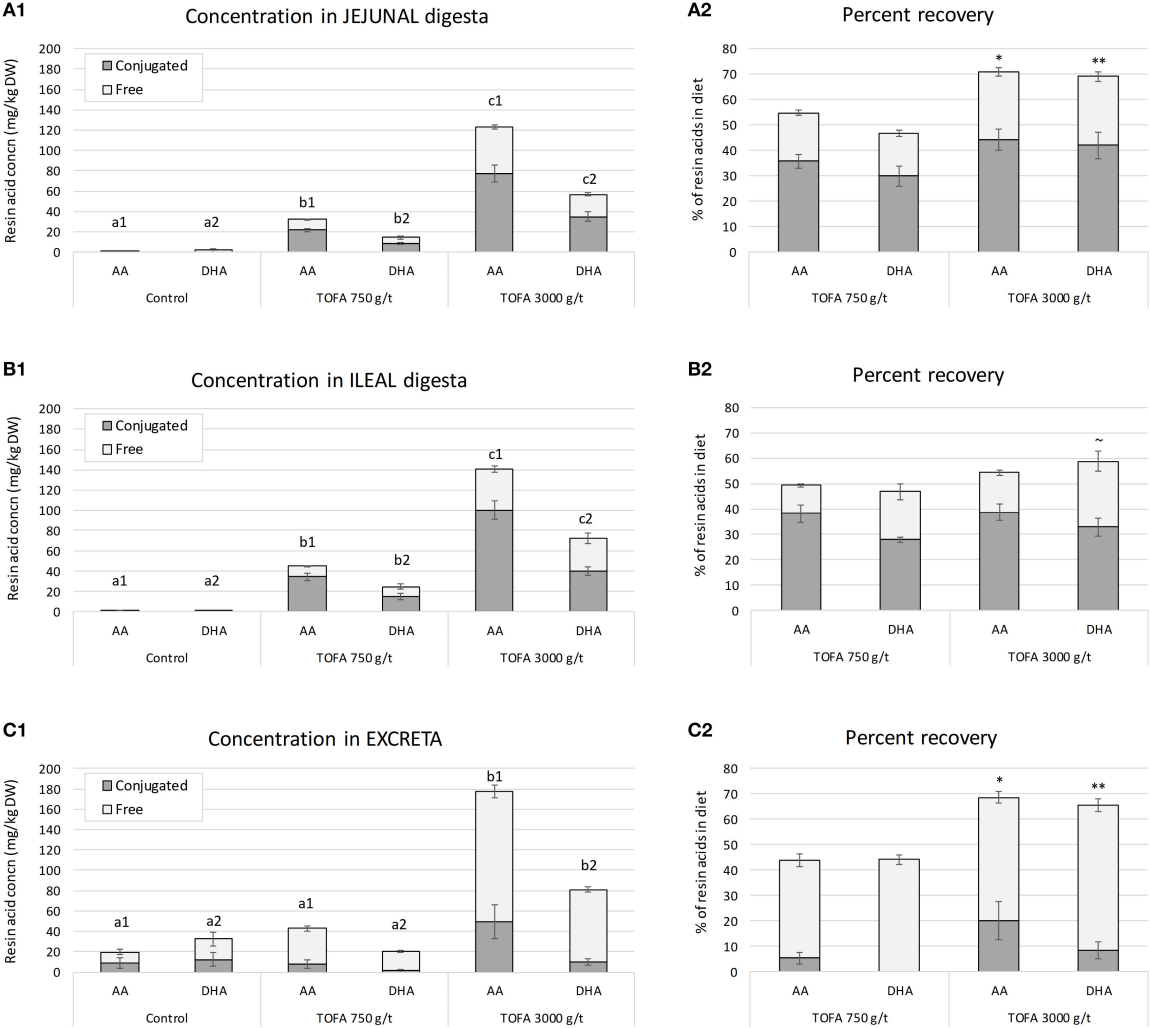
Residual concentration and percent recovery of resin acids in intestinal digesta on day 34. **(A1–C1)** Show the residual concentration, and **(A2–C2)** the percent recovery of resin acids in jejunal digesta, ileal digesta, and excreta, respectively. Total height of bars shows the total abietic (AA) and dehydroabietic (DHA) acid concentration, while the color indicates the proportion of free and conjugated forms. The error bars show standard error of the mean. **(A1–C1)** Different superscripts above the bars for AA (a1–c1) and DHA (a2–c2) indicate significant differences between diets by Tukey HSD test (*P* < 0.05). Recovery indicates proportion of residual resin acids of the amount added in the diet and it was calculated using TiO_2_ as a digestibility marker. **(A2–C2)** Asterisks indicate significance of difference in AA and DHA recovery between the two TOFA-supplemented diets (^~^*P* < 0.1; *P < 0.05; **P < 0.01).

The concentration of resin acids was analyzed in jejunal digesta, ileal digesta, and excreta of 5-week-old broiler chickens. The results showed that the residual concentration of both abietic acid and dehydroabietic acid increased with the increasing rate of TOFA inclusion in the diet (Figure 1). In both jejunum and ileum, the concentration of abietic acid was about twice the concentration of dehydroabietic acid, i.e., close to their respective relative proportions in the diet. In the ileum, the concentrations of both acids were somewhat higher than in jejunum. Assessment of the recovery of in-feed resin acids in samples, using TiO_2_ as an indigestible marker compound, showed that in jejunum, about 50% of dietary abietic and dehydroabietic acids were present in digesta when the dose of TOFA in the diet was 750 g/t. When the dietary TOFA inclusion rate was higher, 3,000 g/t, up to 70% of the two main resin acids were recovered in jejunal digesta (Figure 1A2). In the ileum, the effect of TOFA dose on resin acid recovery was less significant, with the recovery rate ranging from 50 to 60% of abietic and dehydroabietic acid added in the diet (Figure 1B2). Based on the analysis of excreta samples for the main resin acids, it was concluded that the higher the dose of resin acids in the diet, the higher the percentage excreted. When the dose of TOFA in the diet was 3,000 g/t, close to 70% of total abietic and dehydroabietic acids was excreted. When the TOFA dose in the diet was 750 g/t, about 45% of the two resin acids fed was excreted (Figure 1C2).

### Degree of Conjugation of Resin Acids in the Digestive Tract

In order to assess the degree of conjugation, resin acids in digesta were analyzed before and after hydrolytic deconjugation. In digesta sampled from jejunum and ileum, on average 65% of abietic and dehydroabietic acids found were in a conjugated form (Figures 1A,B). In excreta the proportion of conjugated resin acids was lower, representing only 10–20% of total abietic and dehydroabietic acids (Figure 1C).

**Figure 2:**
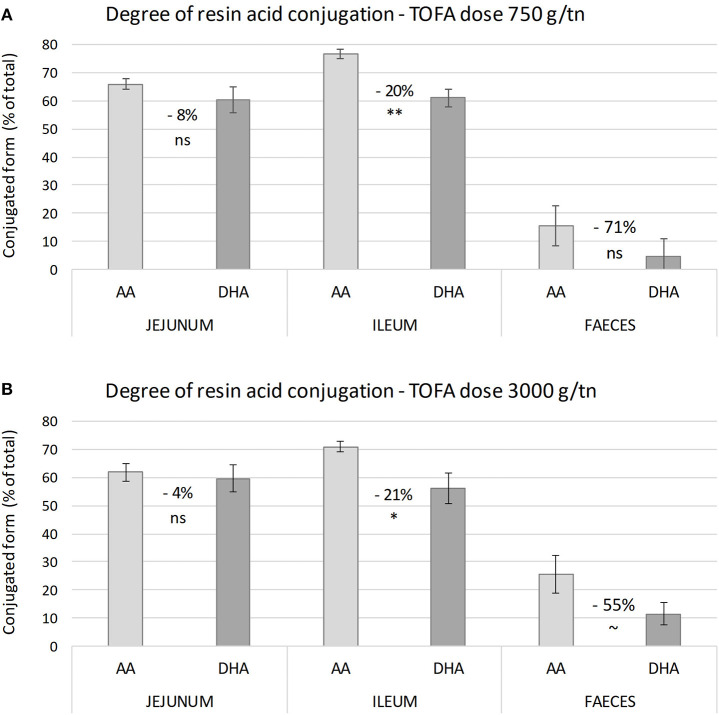
Degree of conjugation of abietic and dehydroabietic acid in intestinal digesta on day 34. **(A,B)** Show the proportion of conjugated resin acids in digesta from different parts of broiler chicken intestine with a tall oil fatty acid inclusion rate in the diet of 750 and 3,000 g/t, respectively. Values between bars indicate percent difference in degree of conjugation between abietic (AA) and dehydroabietic acid (DHA). The error bars show standard error of the mean. Asterisks indicate significance of difference in degree of conjugation (ns, non-significant; ^~^*P* < 0.1; *P < 0.05; **P < 0.01).

The results also revealed a difference in the degree of conjugation of abietic acid and dehydroabietic acid. Analysis of samples representing different parts of the digestive tract showed that abietic acid was consistently conjugated to a higher degree than dehydroabietic acid. The difference between the two acids was statistically most significant in ileum (Figure 2). Numerically, the difference in the degree of conjugation was greatest in excreta, where the proportion of the conjugated form of dehydroabietic acid was less than half that of the conjugated form of abietic acid.

### Free and Conjugated Resin Acids in Liver and Bile

Also in liver and bile samples, both free and total resin acids were analyzed. Overall, the concentration of abietic and dehydroabietic acids in liver tissue was low compared with their concentration in intestinal digesta. In birds on the 3,000 g/t TOFA diet, the concentration of the dominant resin acid, abietic acid, in the liver was below 0.3 mg/kg (Table 4). Furthermore, resin acids were found only after deconjugation, indicating that they were all in conjugated form. The concentration of dehydroabietic acid was less than half that of abietic acid, and thus the relative proportions of the two acids were approximately the same as in the TOFA-supplemented feeds.

**Table 4:** Concentration of free and conjugated resin acids in liver and bile (mg/kg fresh weight) of broiler chickens fed a tall oil fatty acid containing diet for 35 days.

	**Form of resin acid in tissue**	**Control diet**		**TOFA 3,000 g/t**		**SE^a^**		***P*****-value^b^**	
		**AA**	**DHA**	**AA**	**DHA**	**AA**	**DHA**	**AA**	**DHA**
Liver	Free	0.00	0.00	0.00	0.00	0.00	0.00	–	–
	Conjugated	0.00	0.00	0.27	0.11	0.01	0.01	0.0000	0.0000
	Total	0.00	0.00	0.27	0.11	0.01	0.01	0.0000	0.0000
Bile	Free	2.26	1.24	8.41	5.14	0.83	0.46	0.0005	0.0002
	Conjugated	0.00	0.00	35.3	20.1	3.04	1.55	0.0001	0.0000
	Total	2.26	1.24	43.7	25.2	3.45	1.81	0.0001	0.0000

*TOFA, tall oil fatty acids; AA, abietic acid; DHA, dehydroabietic acid*.

a *standard error of the mean*.

b *P-value calculated with students' t-test*.

In bile, the concentration of resin acids was 150-fold higher than in liver tissue, with the total concentration reaching nearly 70 mg/kg. The ratio of dehydroabietic to abietic acid appeared to be somewhat elevated in bile compared with that in liver tissue in birds fed the TOFA-supplemented feeds. Interestingly, some free resin acids were found in bile in birds that were on the control diet. However, nearly 20-fold higher concentration was seen in birds fed the 3,000 g/t TOFA diet (Table 4). In the birds from this dietary group, around 80% of abietic and dehydroabietic acids in bile were in conjugated form.

### Residues of Resin Acids in Other Tissues

Since resin acids are poorly water-soluble, it was of interest to measure their concentration also in intestinal tissue. Jejunal tissue of the TOFA-fed birds indeed had measurable amounts of resin acids, with the sum concentration of abietic and dehydroabietic acids exceeding 3 mg/kg (Table 5). The concentration of resin acids in jejunal tissue of the control birds was below detection.

**Table 5:** Residues (mg/kg fresh weight) of total resin acids in different tissues from broiler chickens fed a tall oil fatty acid containing diet for 35 days.

	**Control diet**		**TOFA 3,000 g/t**		**SE^a^**		***P*****-value^b^**	
	**AA**	**DHA**	**AA**	**DHA**	**AA**	**DHA**	**AA**	**DHA**
Jejunal tissue	0.000	0.000	2.132	1.133	0.104	0.057	0.0002	0.0002
Blood plasma	0.000	0.000	0.311	0.050	0.024	0.005	0.0014	0.0052
Adipose tissue	0.000	0.000	0.000	0.000	0.000	0.000	–	–
Breast meat	0.000	0.000	0.021	0.005	0.007	0.002	0.1747	0.2261

*TOFA, tall oil fatty acids; AA, abietic acid; DHA, dehydroabietic acid; resin acid concentrations represent total acids after deconjugation*.

a *standard error of the mean*.

b *P-value calculated with students' t-test*.

The total resin acid concentration in blood plasma was only one-tenth of that found in jejunal tissue. In adipose tissue, the concentration of resin acids was below detection, while the mean concentration in breast meat was 0.026 mg/kg. However, due to variability in analytical results that were at or just above the detection limit, the concentration of resin acids did not differ significantly between birds in the control group and birds in the group fed 3,000 g/t TOFA for 5 weeks (Table 5).

## Discussion

During the past few decades, the potential of various plant-derived extracts and process byproducts has been tested as feed additives for production animals. TOFA products are a recent example of a feed additive, with several studies demonstrating that TOFA improves the performance of broiler chickens (2–4). More recently, pure resin acids in TOFA have been found to improve performance in the absence of the long-chain fatty acids and other compounds in the raw TOFA byproduct (7). It appears that resin acids play a key role in the positive effects of TOFA, with recent studies showing that pure resin acids decrease collagen-degrading activity in the intestinal tissue (5). Such findings prompted the present investigation on the fate of resin acids, which are presumed to be enduring, in the intestinal tract and tissues of broiler chickens. From a consumer safety and regulatory perspective, it would be preferable if resin acids did not accumulate in edible tissues such as breast meat and liver to any significant extent, but rather become metabolized or eliminated with excreta.

In the present study, a TOFA product containing 8.6% resin acids was added to broiler chicken diets in doses 750 and 3,000 g/t, and birds were fed the diets for 5 weeks. No performance effects were detected, which can at least partly be explained by the high performance in all treatment groups; all groups exceeded the growth performance recommendations of Aviagen for the Ross 308 breed. The total calculated resin acids ingested by an average single bird during the 35-day trial was 272 and 1,087 mg with the 750 and 3,000 g/t dose, respectively. During the last days of the trial, the proportion of in-feed abietic and dehydroabietic acids recovered from feces was ~44 and 67% for diets supplemented with 750 and 3,000 g/t TOFA, respectively. Indeed, the proportion of resin acids that could not be recovered was greater when the dose in the feed was lower, suggesting that the rate of resin acid metabolism or deposition in tissues is rate-limited, not linearly concentration-dependent. There are no prior studies on the fate of resin acids in chickens. In feeding studies with rats given a single oral dose of 2 or 300 mg of tritium-labeled dehydroabietic acid, it was found that 80–90% of the radioactivity was excreted with feces within 2 days (reviewed in 14). In the same study, 5–7% of tritium label administered with dehydroabietic acid was recovered in the urine. In broiler chickens, urine enters the cloaca and is eventually excreted together with feces. Thus, any resin acids secreted from the kidney with urine would have been included in the excreta analysis in this study. Interestingly, our results showed that, at the higher dose of TOFA, the average recovery of abietic and dehydroabietic acids in excreta was 10% higher than recovery in the ileum. This could be explained by the release of urine in the cloaca, distal to the ileum.

When the dose of TOFA in feed was 3,000 g/t, the fate of about 30% of abietic and dehydroabietic acids fed was unknown. If the same proportion also of other ingested resin acids remained unrecovered, about 330 mg of resin acids would be unaccounted for during the 35-day lifetime of an average bird. In the study described here, intestinal tissue, blood, liver, bile, breast meat and abdominal fat were analyzed for resin acids. Based on the results, we estimated that no more than 1 mg/bird was deposited as resin acids in these tissues after the 35-day trial, when TOFA at the 4-fold the recommended dose was fed. Thus, it is likely that the majority of the unrecovered fraction of resin acids was in fact excreted with feces but, prior to that, had been metabolized and was no longer in the original analyzable form.

The results showed that already in the jejunum, only 50–70% of the ingested abietic and dehydroabietic acids could be found. This suggests that resin acid uptake by the bird was highly active already in the upper gastrointestinal tract. Absorption of poorly water-soluble acidic compounds can be expected to occur in the crop and, especially, the proventriculus-gizzard with low prevailing pH. We detected relatively high concentrations of resin acids in jejunal tissue compared with other tissues analyzed. Resin acid concentration in blood was only one-tenth of that measured in intestinal tissue, indicating that liver, and other tissues rapidly remove these compounds from the plasma. Compounds taken up from the intestine enter the portal vein and finally the liver. One of the most important functions of the liver is to render xenobiotic and/or toxic lipophilic compounds harmless and in more water-soluble form. This can be achieved by many different reaction types, such as oxidation, reduction, hydrolysis, or conjugation (glucuronidation and sulfation), the ultimate objective being to facilitate their elimination from the body (18, 19). In the broiler chickens in this trial, the liver appeared to process and pass resin acids to hepatic ducts rapidly, as their residual concentration in liver tissue was low and all resin acids were in conjugated form. The highest concentration of resin acids was measured in bile, of which about 80% was in conjugated form. The fact that also a substantial amount of free resin acids was found in bile suggests that some of these acids were directed to the bile duct unprocessed. The rate of resin acid uptake to hepatic cells possibly exceeded the rate of their processing, leading to resin acid bypass and/or incomplete conjugation. In enterohepatic circulation, the common bile duct leads to the duodenum, and resin acids, together with their conjugates and metabolites, can re-enter the intestinal tract. In the jejunum, distal to the duodenum where bile is secreted, the proportion of total resin acids recovered was at most 70% of ingested in-feed resin acids and, of those, about 65% were in conjugated form. This means that no more than 25% of resin acids in the jejunum could have come directly from the ingested feed, with no prior absorption and enterohepatic circulation through the liver.

It is well-known that intestinal bacteria are capable of hydrolyzing glucuronide and sulfate conjugates, thus potentially enabling re-absorption of drugs from the intestine (20–22). In the present study, the degree of conjugation of resin acids in the jejunum and ileum was the same, indicating that bacteria of the small intestine did not hydrolyze the conjugates to any measurable extent. However, in excreta resin acids were mainly in free form, suggesting that bacteria in the cecum and large intestine actively deconjugated resin acid conjugates. Many aerobic and anaerobic bacteria are not only able to deconjugate, but also to convert resin acids to other compounds (23). According to the results of the present study, the total yield of resin acids did not decrease on moving from ileum to excreta. This suggests that bacteria in the lower intestine of the broiler chickens did not degrade resin acids. Interestingly, a smaller proportion of dehydroabietic acid than abietic acid was in conjugated form in digesta and feces. In bile, no such difference was detected, and therefore there would not appear to be any difference in the rate of resin acid conjugation in the liver. The numerical difference in the degree of conjugation between abietic acid and dehydroabietic acid increased on moving onward in the intestinal tract. This suggests that the apparent difference between the two acids was caused either by a higher rate of deconjugation of dehydroabietic acid by bacteria, or a higher rate of uptake of free abietic acid by the host. Both ways lead to a lower degree of dehydroabietic acid conjugation.

Although the trial conducted was not a toxicity study, it is relevant to review some toxicity studies conducted with resin acids to give a perspective to product doses used in the present study. There are no published toxicity studies for resin acids in broiler chickens. In a 28-day study on mice, 250 mg of abietic acid/kg body weight/day caused no symptoms of intoxication (14). Similar results were obtained in a 15-week study on rats fed 100 mg/kg body weight/day abietic acid (14). Feeding dehydroabietic acid at 25 mg/kg body weight/day also had no adverse effects in rats. In the present study, 21-day-old male Ross 308 broiler chickens were close to 1 kg in weight and with daily feed intake of slightly over 100 grams. Accordingly, the daily intake of total resin acids in birds fed the diet with 3,000 g/t TOFA would be <30 mg/kg body weight. Our results are consistent with those in rodent studies, as the TOFA supplement had no adverse effects on performance. In rodents, LD_50_ values have been determined for abietic acid and dehydroabietic acid, and are reported to be between 2,000 and 4,000 mg/kg body weight in all cases, i.e., ~100-fold higher than the daily intake of resin acids by 21-day-old broiler chickens in the present study fed 4-fold the recommended dose of TOFA (14). It can be concluded that resin acids in the TOFA product used in this study are not likely to cause any adverse effects in broiler chickens.

The ultimate question is whether eating broiler chicken meat produced using a TOFA-supplemented diet poses any risk to human end-consumers. In our analysis of edible tissues such as breast meat and liver, the concentration of resin acids was low even when the diet contained 4-fold the recommended dose of TOFA. Breast meat contained 25 μg/kg of abietic and dehydroabietic acids, in fat tissue resin acids were below detection, and liver contained <300 μg/kg. Thus, in a single meal, a consumer would ingest clearly <100 μg of resin acids, and, at most, only some micrograms/kg body weight. It can be concluded that consumers would not be unintentionally exposed to resin acids to a degree that would pose any health risks. However, it would not be surprising if human health products with resin acids are launched in the near future due to their anti-inflammatory effect and other beneficial effects on intestinal health.

## Data Availability Statement

All datasets generated for this study are included in the article/supplementary material.

## Ethics Statement

Ethical review and approval was not required for the animal study because practices used were not likely to cause pain, suffering, distress or lasting harm equivalent to, or higher than, that caused by the introduction of a needle in accordance with good veterinary practice (EU Directive 2010/63/EU, Chapter I, Article 1, Paragraph 5).

## Author Contributions

KV was responsible for the broiler trial and tissue sampling. KR carried out the resin acid analysis. JA drafted the manuscript. All authors contributed to the design of the study, interpretation of results, editing, and scientific discussions.

## Conflict of Interest

JA, KV, and KR are employed by an independent commercial contract research company Alimetrics Ltd, and HK and JV by a commercial company Hankkija Ltd whose product was used in the present study. This project was also jointly funded by Alimetrics and Hankkija. The funders had the following involvement with the study: Hankkija was interested in knowing the mechanism of action of resin acids in broiler chickens and Alimetrics was interested and had the capability to carry out such research.
